# P41 Evaluation of physician knowledge of tuberculosis across the continuum of care

**DOI:** 10.1093/jacamr/dlag102.047

**Published:** 2026-06-26

**Authors:** S Harshitha, Afrin Siddique, Angel Jennifer, D Suresh Kumar

**Affiliations:** SRIHER, Chennai, Tamil Nadu, India; MMM Hospital, Chennai, Tamil Nadu, India; MMM Hospital, Chennai, Tamil Nadu, India; MMM Hospital, Chennai, Tamil Nadu, India; Best Of IDs, Chennai, India

## Abstract

**Background:**

This study evaluates physician knowledge of tuberculosis across the continuum of care using real-world case–based scenarios. In India, existing evidence shows that many physicians, including interns and residents, demonstrate gaps in diagnostic accuracy and treatment prescribing. However, prior assessments largely rely on theoretical questionnaires and do not reflect decision-making in clinical practice. By using case-based scenarios, this study provides a more realistic evaluation of applied knowledge, aiming to identify clinically relevant gaps and inform targeted educational and antimicrobial stewardship strategies.

**Objectives:**

To assess the knowledge of participating physicians on tuberculosis across real-world clinical scenarios and to identify critical gaps in clinical decision-making.

**Methods:**

This cross-sectional study was conducted in March 2026 as part of a World Tuberculosis Day awareness CME via a structured Zoom webinar involving physicians managing tuberculosis in clinical practice. Knowledge was assessed using 20 real-world, case-based scenarios, each with five response options, delivered as live polling questions. Cases were developed by infectious disease specialists and validated by an expert panel. Responses were anonymized and extracted from the Zoom platform. Descriptive statistics summarized response patterns and scores. Knowledge levels were categorized using predefined thresholds, and comparative analyses were performed using chi-square or Fisher’s exact test, with *P*<0.05 considered statistically significant.

**Results:**

Of 180 registered physicians, 130 participated in the CME-based assessment. Across 20 case-based questions, the number of respondents per question ranged from 80 to 100, with a mean of 90.8 participants. The overall mean correct response rate was 36.8% (SD 23.4). A total of 6/20 (30%) questions achieved ≥50% correct responses, while 14/20 (70%) had <50% correct responses. Domain-wise analysis demonstrated variability in correct response rates across the tuberculosis care continuum.

**Conclusions:**

Only 30% of scenarios achieved ≥50% correct responses, indicating significant gaps in physician tuberculosis knowledge. Webinar-based design limits generalizability. Structured, real-world TB case–based education should be prioritized to improve clinical decision-making.

